# The process of developing dynamic capabilities: The conceptualization attempt and the results of empirical studies

**DOI:** 10.1371/journal.pone.0249724

**Published:** 2021-04-29

**Authors:** Szymon Cyfert, Anna Chwiłkowska-Kubala, Witold Szumowski, Radosław Miśkiewicz

**Affiliations:** 1 Department of Organization and Management Theory, Poznań University of Economics and Business, Poznań, Poland; 2 Department of Organization and Management Theory, Wrocław University of Economics and Business, Wrocław, Poland; 3 Faculty of Organization and Management, Silesian University of Technology, Gliwice, Poland; Universitá Cattolica del Sacro Cuore, ITALY

## Abstract

While most researchers interested in the concept of dynamic capabilities focus their attention on analyzing how companies transform their resources to compete in their environment, the process of developing dynamic capabilities is treated as a marginal issue. Although the literature suggests various approaches to developing dynamic capabilities, they are formulated in general terms, and doubts can be raised about the links between actions. There is also a lack of empirical research indicating the links between activities in the process of developing dynamic capabilities and their influence on the effectiveness of an organization. The aim of the study is to formulate a proposal for a model of the process of developing dynamic capabilities aimed at increasing the economic effectiveness of a company and to determine the links between the activities in the model. The theoretical contribution of the paper consists in presenting a model of the process of developing dynamic capabilities aimed at increasing the economic effectiveness of the company. The results presented in the paper refer to an empirical examination of the model of developing dynamic capabilities, covering five activities: searching for opportunities; knowledge management and learning; coordination; configuration and reconfiguration; and organizational adaptation. The study also includes an examination of the possible impact the components of the dynamic capabilities building process have on a company’s performance. The study uses the survey method and data was obtained from top managers. The conclusion, based on data from 471 Polish companies, was made using structural equation modelling. The results of the empirical research suggest that the individual activities in the process of developing dynamic capabilities are interconnected, and through mutual interactions and couplings, they positively affect the economic effectiveness of an enterprise. The results indicate that searching for opportunities is the precursor, and the main factor influencing the other activities in the process, which suggests that managers should focus on improving activities in this area.

## Introduction

The attempts to explain the differences in performance among companies (also within the same industry) have been a cause for discussion in the category of dynamic capabilities proposed by the Teece, Pisano and Shuen research team [1]. The dynamic capabilities concept has its origins in the resource approach and the resulting deliberations on the various categories of enterprise capabilities [2]. Referring to the concepts of key competences [3], strategic resource characteristics [4], and the importance of organizational capability categories [5], D.J. Teece, Pisano, and Shuen have undertaken reflections on the capabilities that are involved in reconfiguring the resource base to adapt to changing environments. The Dynamic Capabilities approach therefore focuses on both the internal perspective of the organization (modifying the layout of resources, broadly defined) and the external perspective (orientated towards adapting to and following a dynamically changing environment) [1].

The approach on the perception of dynamic capabilities by Teece, Pisano and Shuen [1] was discussed by Eisenhardt and Martin [6]. These two articles have provided the basis for a scientific reflection on the issue of dynamic capabilities. A comparison of these two proposals regarding the perception of dynamic capabilities results in a different perception of their importance for the success of an enterprise. Although both proposals point to the importance of organizational routines and processes, Eisenhardt and Martin attribute more importance to the reconfiguration of resources than to dynamic capabilities in the success of a company [7].

As a consequence of the ever-growing interest in the issue of dynamic capabilities, there have been various attempts to conceptualize them. For example, dynamic capabilities are seen as higher-order capabilities [8, 9]; as referring to the category of routine [10, 11]; or, in general, are seen as supporting activities aimed at deliberately reconfiguring the resource base [12–14]. The discussion on dynamic capabilities focuses more on what dynamic capabilities are [6, 15–21], and how they differ from operational capabilities [22–26], than on the question of how they are developed [7, 27, 28].

Criticism of the ambiguity of the definition and the complexity of the construction of dynamic capabilities [29], is countered not only by broad theoretical considerations, but also by a differentiated research approach to the issue of dynamic capabilities [2]. On the basis of the conducted considerations, a conclusion emerges that dynamic capabilities enable the creation of new, exceptional, valuable and difficult to follow configurations of resources and organizational changes, thanks to which an organization has a chance to adapt to changing operating conditions [30].

The concept of dynamic capabilities emphasizes the key importance of this category of capabilities in adapting to changes in the environment [1]. On the other hand, however, the considerations on the category of dynamic capabilities are accompanied by criticism, indicating that dynamic capabilities are important from the perspective of resource allocation, but in a high velocity environment their role in creating an organization’s adaptability is not critical [6]. The discussion on the essence of dynamic capabilities and their participation in strategic adaptation capabilities is still open [31].

The issue of developing dynamic capabilities is all the more important because developing and embedding dynamic capabilities in a company’s management processes can identify and prevent harmful forms of path dependence, avoid blockages and maintain levels of competence building and organizational changes adequate in terms of market evolution or management [32]. This state of affairs, which translates into problems with the practical use of the demands regarding the concept of dynamic capabilities [33], especially in relation to organizational effectiveness [10, 34–36], encouraged us to propose and further test a model for the process of developing dynamic capabilities, affecting the economic effectiveness of an organization.

The aim of this research is to investigate the process of developing dynamic capabilities with a view to the category of economic effectiveness and therefore answer four research questions:

1. What are the activities that make up the dynamic capabilities development process?
2. What elements are included in the scope of individual activities in the process of developing dynamic capabilities?
3. How are the connections between individual activities in the process of developing dynamic capabilities?
4. To what extent do individual activities in the process of developing dynamic capabilities affect the economic effectiveness of an organization?

Our paper has been organized as follows. First, on the basis of a literature review, we propose a five-stage model for the process of developing dynamic capabilities and, in an attempt to operationalize it, we identified 27 components. Secondly, we discuss the relationships between dynamic capabilities, performance and effectiveness. Thirdly, referring to the results of a critical review of the literature, we present the research methodology. In the next section, based on the results of the research, we conduct a discussion referring to the relationships between activities in the model regarding the process of developing dynamic capabilities; also indicating the relationship between activities and economic effectiveness. In the summary of the study we define the managerial implications and research limitations, allowing us to set the directions for further research.

### The process of developing dynamic capabilities

Zahra, Sapienza and Davidsson [14] indicate that dynamic capabilities are formed under the influence of various variables, both subjective and objective, so that having dynamic capabilities does not necessarily lead to better results per se. For a dynamic capability to achieve the expected benefits and provide a basis for building a sustainable competitive advantage [37–41], it must be properly targeted and implemented, which indicates the importance of the dynamic capability process [10, 31, 42]. The need for a systemic and process approach to the management of dynamic capabilities also results from the cost of activities related to their development and use, as well as their impact on the functioning of an organization both in the short and long term, which may translate into profits [16].

Eriksson [43] notes that in the literature on the subject, dynamic capabilities are treated in terms of processes whose dynamic nature is related to their impact in time. Some authors [12, 33, 44] indicate that dynamic capabilities include both organizational and managerial processes aimed at identifying the needs or opportunities for change and making changes. Collis [5] emphasizes that dynamic capabilities influence the pace of change regarding ordinary capabilities, taking into account dependency paths and market positions; as also indicated by the studies of Eisenhardt and Martin [6], which assume that dynamic capabilities influence the organizational and strategic procedures that underpin resource based decisions.

In the discussion on the essence of the process of developing dynamic capabilities, Zollo and Winter [11] indicate that dynamic capabilities are derived from learning processes: the accumulation of experience, the articulation of knowledge and the codification of knowledge, and that they relate to specific and identifiable processes related to the integration, reconfiguration, acquisition and release of resources. A similar set of activities in the dynamic capability development process is indicated by Teece, Pisano and Shuen [41]; assuming that the dynamic capability development processes include coordination, integration, learning and reconfiguration activities; which in turn are studied by some dynamic capabilities researchers [eg. 45, 46]. Referring to the Teece concept, Pisano and Shuen, Ambrosini and Bowman [16] present an impact model for the dynamic capability process, assuming that dynamic capabilities have a direct impact on a company’s resource base and involving four activities in the dynamic capability process: reconfiguration, use, learning and creative integration.

Embedding a reflection on dynamic capabilities in the area of knowledge, Zollo and Winter [11] formulate a proposal for a ‘knowledge evolution cycle’ model that represents a development of dynamic capabilities and operational procedures. In the model, describing the changes in the course of actions leading to a higher level of annuities, they distinguish four actions: change, selection, replication and retention. The retention activities lead to changes in organizational routines and attitudes, which may initiate the next cycle of change. Based on the Zollo and Winter model, Cepeda and Vera [22] present a proposal for a knowledge-based dynamic capabilities model; which starts from the level of strategic decisions, treated in terms of dynamic capabilities; thus acknowledging the links between dynamic and operational capabilities.

Teece [47] assumes that dynamic capabilities are developed in the following processes: (a) detecting and shaping opportunities and threats; (b) using capabilities based on the choice of product architecture and business models, outlining organizational boundaries, defining decision-making rules and building employee loyalty; and (c) maintaining a level of competitiveness by strengthening, integrating, protecting and, if necessary, reconfiguring intangible assets.

### Conceptualization of the dynamic capabilities development process

Referring to the models described, making an assumption about the importance of knowledge management [27, 48–53] and the connection of dynamic capabilities to environmental phenomena [10, 46, 54–58], we propose a five-stage model of developing dynamic capabilities, aimed at increasing the effectiveness of an organization, which consists of (a) searching for opportunities; (b) knowledge management and learning; (c) coordination; (d) configuration and reconfiguration; and (e) organizational adaptation. The model assumes that the individual activities in the model are interconnected and interact with each other through direct and indirect links; and referring to the view cited in the literature that one of the basic conditions for an organization to exist and ensure its long-term sustainable success is the ability to renew its sources of competitive advantage [4], we assume that the process of developing dynamic capabilities, due to the nature of dynamic capabilities, should not be treated as a one-off, passive action, constituting an ex post reaction of the organization to changes in the environment, but should be a process allowing for the anticipation of change.

### Searching for opportunities

The implementation of activities undertaken at the stage of searching for opportunities is based on the use of mechanisms for monitoring the environment and the needs of stakeholders [59], which allow for the identification of the processes of changes in the environment, constituting the basis for searching for opportunities [56], as well as the capture of weak signals in the environment [33], the assessment of the correctness of developing the set of dynamic capabilities used [60], the change of employees’ attitudes and the generation of a set of options for potential dynamic capabilities [32, 60, 61], on which managers’ attention should be focused. The model assumes that the dynamic capabilities associated with searching for opportunities include:

- analysing trends and phenomena in the environment, aimed at creating new customer needs and anticipating the actions of competitors [62];
- creating new ideas to mobilize participants and lead to changes in their attitudes [63];
- identifying the needs for change as a result of phenomena occurring in the environment and the organization’s potential [1].

### Knowledge management and learning

The literature points to their being a relationship between knowledge management and dynamic capabilities [7, 11, 28, 33, 60, 64], emphasizing the key importance of dynamic capabilities for the acquisition, creation, absorption, distribution and reconfiguration of knowledge for the processes of competitive advantage. Organizational knowledge, developed by learning processes, is perpetuated in new ways of doing things, new organizational routines or new logic in the functioning of an organization [40]; which are difficult to replicate [33]. Zollo and Winter [11] prove that dynamic capabilities are de facto the result of organizational learning [14] and established ways of collective activity, through which an organization systematically generates and modifies its operational behaviour [65] in a way that increases its level of effectiveness. The learning activities, which are a source for the dynamic capability development processes, provide the basis for selecting the optimal set of dynamic capabilities in the next stages. Although the chronology of activities adopted in the model may seem to be contradictory to the logic of efficient operation [66], it is possible to indicate premises justifying the proposed sequence. Applying a different mode of proceeding, in which knowledge acquisition preceded searching for opportunities, would lead to the phenomenon of information redundancy, forcing an organization to bear the costs of acquiring and gathering information that the organization would not be able to use; which from a business point of view would be difficult to justify. Since knowledge in the dynamic capabilities development process is the result of social activities and the efforts of an organization’s participants [67], it is important to link the knowledge management processes with the coordination activities that allow for adjustments to be made at the individual stages of the dynamic capability development process. Dynamic capabilities that are related to knowledge management and learning include the following:

- acquisition of knowledge, which is the basis for transformational activities in the area of dynamic capabilities [33];
- knowledge transfer within the organization, ensuring proper information supply to the units participating in the processes of organizational change [68];
- allocation and storage of knowledge within an organization, allowing for the proper embedding of knowledge in organizational units and ensuring synergistic potential as a result of knowledge accumulation [69];
- intellectual property management, ensuring an adequate level of protection for an organization’s property rights [70];
- encouragement of employees to experiment, which is the basis for the emergence of new, breakthrough ideas [33].

### Coordination

An organization’s success in developing dynamic capabilities depends on resource management [69, 71–73], which is implemented through coordination activities; with resources, applying the concept of outsourcing, not necessarily owned by the organization [74]. It should be assumed that as important as activities related to acquiring and creating new resources and skills are, activities related to the disposal of resources and skills, which, from the point of view of dynamic capabilities building processes, can be a long-term burden for an organization. Redundancy of resources and skills, due to the need for an organization’s involvement, may lead to a reduction in the level of flexibility in an organization’s functioning, which from the point of view of the dynamics of the environment is an undesirable phenomenon. Within the area of coordination, dynamic capabilities include the following:

- creating a vision that integrates stakeholders, clearly indicating the actions implemented for stakeholders by organizations and the values created for them [59];
- building stakeholder loyalty as a basis for securing key resources in all dimensions of an organization’s operations [75];
- integrating activities in the supply chain, providing the basis for determining the value created in the chain and enabling the capture of that value [76];
- managing strategic alliances to acquire and secure key resources and ensure a high level of quality in external activities [56];
- building the commitment of employees, allowing them to be involved in the activities of an organization and increasing the level of their involvement [77];
- integrating and coordinating business processes, supporting processes of change and ensuring an appropriate level of organization cohesion [78].

### Configuration and reconfiguration

The defined optimal set of dynamic capabilities will remain an abstract description of an organization’s development opportunities until the right conditions are created for its implementation [79, 80]. Visionary leaders should refer to internal and external stakeholders when taking action to change dynamic capabilities [81–83] so that they believe in the reality of an organization’s vision and engage in processes of change [84]. Those responsible for the dynamic capabilities development processes must be aware of the importance of emotional stakeholder engagement for the long-term success of an organization [83, 85, 86]. This means that they should coordinate their activities within an organization in such a way that stakeholders will not only accept the processes of change, but will be confident in the effectiveness of their implementation, trust the decisions of managers and believe in the wisdom of the actions undertaken. Activities in the area of configuring dynamic capabilities; due to the need to involve diverse, key stakeholders in an organization; are iterative in nature, causing the defined model of dynamic capabilities to be modified. In the course of each iteration, a kind of negotiation game takes place between the people participating in the definition of dynamic capabilities, aimed at defining a set of actions integrating entities interested in an organization’s success. Dynamic capabilities in the area of configuration and reconfiguration include the following:

- creating resources and skills, critical to the development of an organization, that are not available in the organization’s environment, or where acquisition costs are higher than the costs of production [87];
- acquiring resources and skills [88];
- integrating resources and skills to leverage the synergistic potential that an organization can have [1];
- creating innovations that enable the right dynamic capabilities to build a foundation for a competitive advantage [89];
- disposing of (releasing) unnecessary and redundant resources and skills that will generate limited added value from an organization’s long-term growth perspective [90];
- implementing new technologies that provide the right conditions for the functioning and development of an organization [63].

### Adaptation

The literature indicates that the management systems of companies using the concept of dynamic capabilities should be characterized by a high level of decentralization [91–93], providing flexibility and allowing a rapid response to changes in the environment. This is also the view expressed by Volberda and Elfring, according to which developing dynamic capabilities requires the use of an adhocratic structure, in terms of Mintzberg, or an organic structure [94]. The implementation of the adaptation process makes it possible to ensure that system solutions are adapted to changes in the environment [95] and focus on signals from the environment [60], which is the point of transition to the next cycle of building dynamic capabilities. Dynamic adaptive capabilities include the following:

- transformation of the business model, and by indicating the logic of an organization’s operations, reflecting a modified arrangement of strategic objectives and the trajectory of dynamic capabilities of an organization [46];
- management of an organization’s boundaries, within which the dimensions of effectiveness, power, competence and organizational culture are transformed [96];
- assurance of an appropriate level of dynamism in the strategic management process, taking into account the need to influence the inside of an organization in such a way as to achieve an optimal level of alignment with the environment, while maintaining the identity of the organization [97];
- improvement of an organization, in the framework of which comprehensive actions are taken to integrate and change the solutions used in an organization, ensuring a higher level of coherence between an organization and its environment [98];
- adaptation and implementation of best management practices [99];
- assurance of the flexibility of the organizational structure, which is related to the implementation of modern solutions based on organizational networking;
- management of the identity of an organization.

### Dynamic capabilities in relation to performance and effectiveness

Since the beginning of the discussion on the category of dynamic capabilities, a thesis has been put forward that there are certain correlations between dynamic capabilities and the broadly understood results of companies [1].

In the course of the discussion, an observation emerges that the value of resources appears when they are properly configured and integrated to create the capacity of an organization [100]. It is worth noting that the reconfiguration of resources alone is not a guarantee of improving a company’s results. The key importance is attributed to the optimal, from the perspective of a given entity, reallocation of resources [101]. Attempts to determine the importance of dynamic capabilities in achieving various company results are justified by the different characteristics of available resources and the capabilities of a given company [102].

Research on the impact of dynamic capabilities on the performance of enterprises is related to the different perspectives of their operations as well as the different categories of results. For example, Kareem and Mijbas [35] suggest that dynamic capabilities are involved in human resources development and thus determine an organization’s effectiveness, which includes not only influencing an organization’s human resources development processes, but also by directly influencing its performance. Darawong [103], on the other hand, points to the direct impact of such dynamic capabilities as sensing, learning and integrating on the effectiveness of the project team.

An analysis of the literature shows that the discussion on the importance of dynamic capabilities in generating profit is developing in different directions. On the one hand, the literature [104] points to a growing number of studies showing the indirect nature of the impact of dynamic capabilities on a company’s performance. On the other hand, however, an analysis of the literature on the subject also provides conclusions supporting their direct impact [105, 106]. Studies indicating the direct impact of dynamic capabilities on the performance of enterprises point out that dynamic capabilities intensify the benefits of engaged resources and lower-level capabilities [107].

The moderating nature of dynamic capabilities is highlighted by the Wang research team [53]. Based on the results of research on the impact of information technology on performance, the researchers indicate that, in the end, it is the dynamic (knowledge-based) capabilities (their different levels/engagement), as moderating variables, that determine the results achieved by a company. The indirect impact of dynamic capabilities on an enterprise’s performance is indicated by the results of the studies conducted by Bhatt and Grover [108], which relate dynamic capabilities to learning processes. Researchers suggest that the indirect impact of dynamic capabilities results from the fact that the learning processes are translated into results when involved in specific projects. In turn, the Battisti and Deakins study [109] concludes that with higher managerial and/or entrepreneurial skills, an organization is better able to deal with rapidly changing environmental conditions (including extremely negative ones such as natural disasters).

A similar direction is taken in the research prepared by Drnevich and Kriauciunas [110], which indicates that dynamic capabilities change organizational processes, but do not directly affect a company’s performance. Moreover, Drnevich and Kriaciunas suggest that the lack of, or even negative impact on, results at the level of the organization as a whole may result from an underestimation of the importance of dynamic capabilities or their incompetent use. However, on the other hand, researchers emphasize that capturing the results of engaging dynamic capabilities from the perspective of the results of the whole organization is a challenge for the researchers: both temporal (the need for research in the long run) and methodical (the problem of capturing the effects of applying dynamic capabilities).

When considering the importance of dynamic capabilities in the development of a company’s results, it is important to take into account the limited possibilities of examining this phenomenon, resulting from the complexity of creating and engaging dynamic capabilities. The impact/effects of dynamic capabilities on a company’s performance cover many areas of activity [53, 111]. The research suggests that the impact of dynamic capabilities on a company’s performance in a broad sense depends on the context of the company’s operations, both internal and external. As a result of the research on the nature of dynamic capabilities, observations emerge in line with Eisenhardt and Martin [6] that the role and nature of dynamic capabilities in the performance of a company are conditioned by the level of environmental variability [112, 113]. In turn, the meta-analysis of the research team Fainshmidt et al. [30], indicates that there is a correlation between dynamic capabilities and the results of companies, and this correlation is stronger in conditions of dynamic changes in the technological environment.

## Methodology

### Sample selection and description of the research tool

The formulation of a model proposal for the process of developing the dynamic capabilities of an organization and determining the scope of their impact on the economic effectiveness of the organization required to carry out a four-phase procedure.

Firstly, in order to propose the variables of the survey, we conducted an in-depth literature review, which allowed us to propose a list of 27 dynamic capabilities assigned to a five-step model of developing dynamic capabilities.

Secondly, we asked a panel of 15 experts (consisting of eight practical representatives (CEOs) and seven representatives of the academic community) to make suggestions regarding the proposal to be formulated. As Table 1 shows, in the end the following types (categories) of dynamic capabilities and their components were suggested.

**Table 1:** Categories of dynamic capabilities studied in the research procedure.

Categories of dynamic capabilities		Components of dynamic capabilities
I	Searching for opportunities	• ability to analyze the environment aimed at creating new customer needs, • anticipating the actions of competitors, • ability to create new ideas and • awareness of changes in the environment.
II	Knowledge management and learning	• acquiring knowledge • transferring knowledge within the organization, • allocating and storing knowledge, • managing intellectual property, • encouraging innovation and experimentation
III	Coordination	• creating a vision that integrates stakeholders, • building stakeholder loyalty, • integrating activities in the supply chain, • managing strategic alliances, • building employee engagement, • creating consistent decision-making rules, • integrating and coordinating business processes
IV	Configuration and reconfiguration	• creating resources and capabilities, • acquiring resources and capabilities, • integrating resources and capabilities, • creating innovation, • disposing of (releasing) unnecessary and redundant resources and capabilities • implementing new technologies
V	Adaptation	• transforming the business model, • managing the organization’s boundaries, • ensuring a dynamic strategic management process, • improving the organization, • adapting and implementing best management practices, • ensuring the flexibility of the organizational structure, • managing the organization’s identity.

Source: own work.

Moreover, the respondents were asked to assess the economic effectiveness of the organizations they managed compared to their competitors, taking into account the following aspects: employment growth, sales growth, market share growth, profitability dynamics, and customer loyalty level. These categories were selected based on a literature overview regarding perspectives on organizational performance [e.g. 69, 114].

Determining the components that make up each category of dynamic capabilities was the basis for constructing a survey. The cover letter for the survey provided respondents with confidentiality and anonymity, giving them the opportunity to submit the survey without providing their name or company name. To avoid any misunderstanding, an additional attachment explained all the terms used.

For several reasons in the study conducted online, a structured questionnaire was used to collect primary data. First of all, it resulted from the assumed research goal, which required quantitative research, indicating the links between the activities in developing dynamic capabilities. Secondly, in order to understand the activities that are part of the process of developing dynamic capabilities, we wanted to formulate conclusions that had the characteristics of generalizations.

Thirdly, in order to check the communicativeness of the questionnaire a pilot study was carried out, the results of which did not modify the list of dynamic capabilities except in the way the questions were formulated (so that they were more understandable for the respondents); and for the proposed scales (since the variables of interest in the proceedings cannot be obtained from organizational documentation (reports or financial statements), we used perception measures on a Likert five-stage scale from 1 (strongly disagreeing) to 5 (strongly agreeing)). The results of the pilot study also determined the method of selecting the target group of respondents. As the respondents indicated that in a situation where they did not have the appropriate knowledge, they could have problems with filling in the questionnaire, it was decided the relevant research was to be limited to respondents meeting two criteria jointly (a) being postgraduate or MBA students at Poznań University of Economics and Business and (b) being CEOs or being members of top management. Additionally, due to the need to assess the phenomena occurring over time, enterprises existing for less than five years were excluded.

Fourth, using the CAWI (computer-assisted web interview) method, we asked respondents who met the above-mentioned conditions to make an assessment of the dynamic capabilities of the organizations they managed. In the research proceedings, assuming the company as an analytical unit, a single respondent project was used [115]. In order to ensure an appropriate sample size and thus the possibility of generalization, we did not focus on a specific sector but addressed invitations for filling in the questionnaire to managers managing companies operating in Poland, assuming that the Polish economy, due to its growth dynamics (the highest average GDP growth in the last 30 years), is an appropriate context for conducting research on dynamic capabilities.

Invitations to complete the survey were sent to 730 respondents. In total, we received 539 completed questionnaires, out of which 86 questionnaires were rejected due to a lack of complete answers (57 respondents did not answer all the questions) or failure to meet the age criterion (29 respondents indicated that their companies had existed for less than 5 years), resulting in a sample of 471 companies. Table 2 presents the characteristics of the surveyed enterprises.

**Table 2:** Characteristics of the surveyed enterprises (n = 471).

**Type of business activity**	
Production	28%
Services	13%
Trade	11%
Transport and logistics	10%
Financial and insurance services	10%
Construction	8%
Gastronomy and hotel industry, recreation	7%
Municipal and public sector	4%
Farming	4%
Health care	3%
Computer and communication technology	3%
**Organization’s Age**	
5–9 years	22%
10–19 years	35%
20–29 years	26%
more than 30 years	16%
**Number of employees**	
10 do 49	44%
50–249	23%
more than 250	33%

Source: own study

### Research limitation

When analysing the course of the conducted research, it is necessary to indicate its potential limitations. One of the most important ones is the approach based on a single respondent project. The authors are aware of the limitations of its application [116], however, it was assumed that it is more valuable to obtain information from one respondent having comprehensive knowledge of the organization than from many respondents having specialist but fragmented knowledge. Moreover, the applied approach enabled a relatively large amount of data to be obtained, which assuming a method based on an analysis of organizational and financial documentation, would not have been possible both due to a very high workload and the barriers related to potential company secrets. Moreover, it should be noted that the respondents were managers managing organizations (CEOs); therefore, are people who are best oriented both in terms of their dynamic capabilities and have a very good overview of the economic efficiency of the organization.

Nevertheless, one should be aware of the potential limitations resulting from the subjectivity of assessments (especially in the area of assessing the economic effectiveness of an organization against competitive entities), but the results of Protogerou et al. indicate a high correlation between subjective and objective measures of the variables [117]. The sample size is also a limitation, since for a confidence level of 0.95, a sample size of 471 and an assumption of infinite population size, the maximum statistical error was 4.52%.

## Research results

In order to determine the relationship between individual categories of dynamic capabilities and to determine their impact on economic effectiveness, synthetic indicators for individual types of dynamic capabilities and economic effectiveness were created, based on the arithmetic mean of individual components within the category. For each of the indicators the test reliability coefficient values were calculated. Assuming the value of Cronbach’s Alpha coefficient at a level of 0.6 [118] as the limit for newly created measurement scales, it was necessary to correct the components of one of the indicators–the configuration and reconfiguration index (originally the alpha value was 0.59). After limiting the components of the indicator to creating resources and capabilities, acquiring resources and capabilities, and integrating resources and skills, the alpha value for this indicator increased to 0.65. The alpha values of the indicators used for the purpose of the conducted analyses are presented in Table 3.

**Table 3:** Values of the Alfa Cronbach test reliability coefficient for individual indicators.

Indicator	Alpha value	Variable symbol
Searching for opportunities	0,73	*S*
Knowledge management and learning	0,80	*K*
Coordination	0,65	*C*
Configuration and reconfiguration	0,65	*R*
Adaptation	0,69	*A*
Dynamic capabilities (HOI)		*DC*
Economic effectiveness	0,78	*EF*

Source: Own study

**Fig 1:**
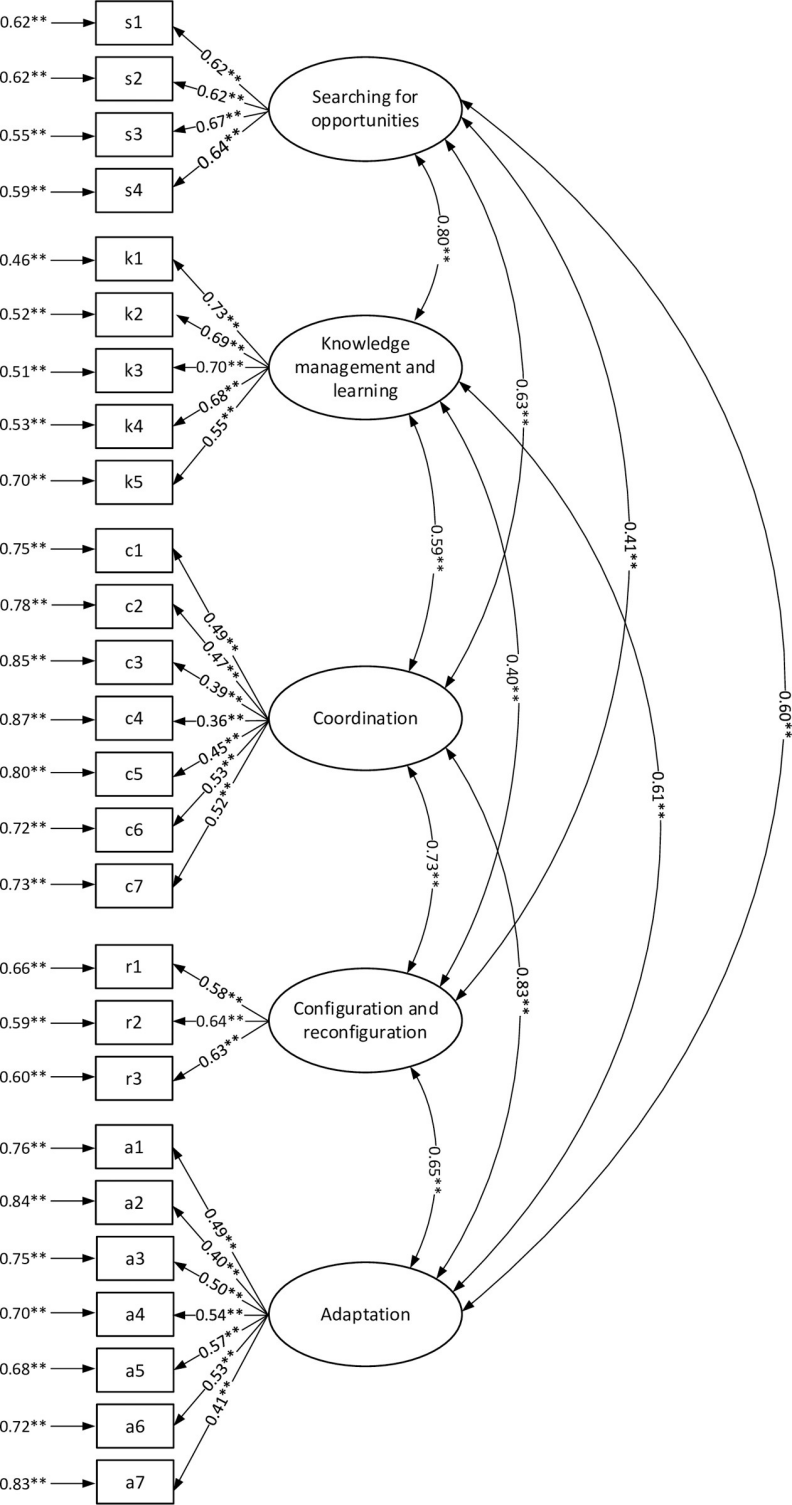
Confirmatory factor analysis for the measurement model of dynamic capabilities (factor loadings error variance and correlation coefficients). Source: Own study.

An exploratory factor analysis was also carried out for all the variables. The application of the Kaiser criterion and additionally the settlement diagram enabled the formulation of a statement about the proper construction of indicators due to their one-factorial character. Next, the authors conducted a confirmatory factor analysis for each measurement model indicator (independent for dynamic capabilities and for economic effectiveness). The results of these analyses (Fig 1) show that the model of dynamic capabilities fits well with the data and empirical values; the GFI, SRMR, NFI, CFI, and IFI values being respectively: 0.935, 0.041, 0.946, 0.952, and 0.952.

**Fig 2:**
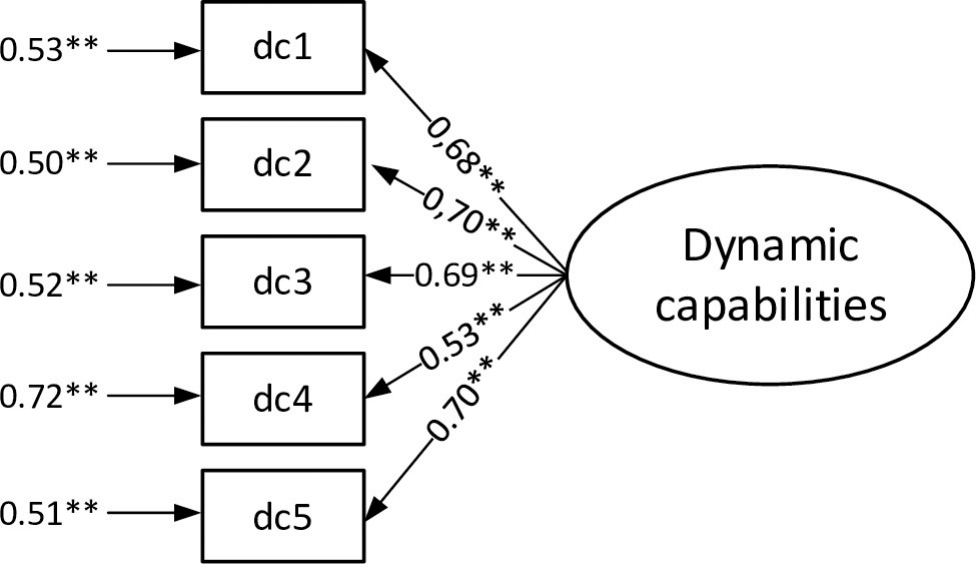
Confirmatory factor analysis for the measurement model of dynamic capabilities for the higher order indicator (factor loadings and error variance). Source: Own study.

Moreover, the values of the correlation coefficients between individual indicators in the confirmatory analysis indicate that it is possible to create a higher order indicator [119, 120] describing the process of developing dynamic capabilities. Therefore, in the next step, such an indicator was proposed (based on the average value of lower-order indicators). The results of confirmatory factor analysis of the measurement model for the higher order indicator of developing dynamic capabilities are shown in Fig 2. For this analysis the GFI, SRMR, NFI, CFI, and IFI values were respectively: 0.928, 0.063, 0.886, 0.891, and 0.892.

**Fig 3:**
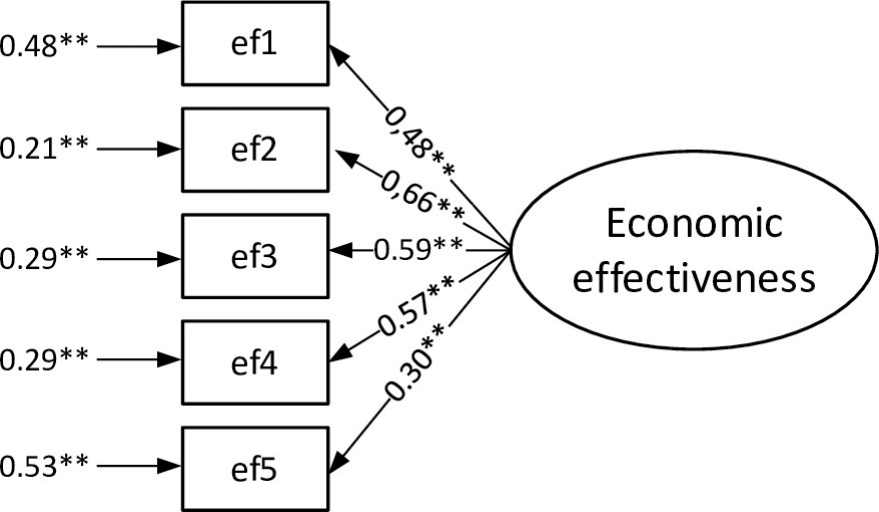
Confirmatory factor analysis for the measurement model of economic effectiveness (loadings and error variance). Source: Own study.

For the economic effectiveness measurement model the results of confirmatory analysis are shown in Fig 3, and for this model the GFI, SRMR, NFI, CFI, and IFI values were respectively: 0,995, 0.017, 0.991, 0.999, and 0.999.

In the next step in the proceedings we used an exploratory approach to path analysis aimed at determining the relationships between individual variables in order to find an answer to the question of the relationships between specific activities in the dynamic capability development process.

**Fig 4:**
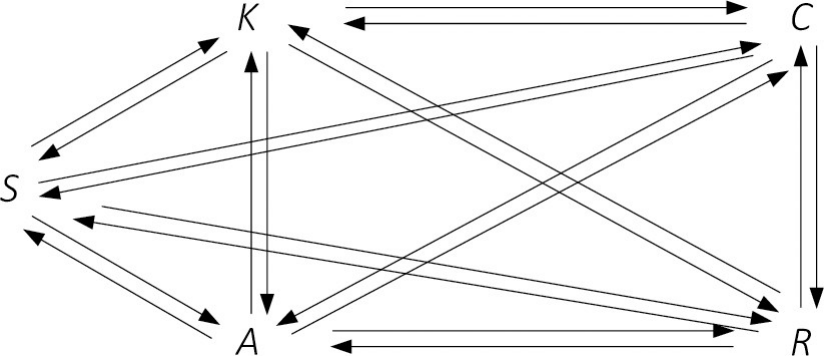
Primary model for variable link analysis. Source: Own study.

The authors of the study, when starting the analysis, took into account all hypothetical causal relationships between the variables, which are dynamic capabilities (Fig 4).

With such a large number of paths in relation to the size of the test sample, it was impossible to obtain satisfactory p-values of chi-quadrate statistics. It was also impossible to determine the GFI SRMR, NFI, CFI, and IFI values. Therefore, based on the obtained results, the values of the parameters (which describe relationships between variables) that assumed statistically insignificant values were gradually rejected. This action was repeated, eliminating the individual paths until all the assumed model fitting measures reached their preferred values, and all model parameters for each path were verifiable. Table 4 presents the steps of eliminating the paths leading to the development of the final model.

**Table 4:** Steps of eliminating paths in SEM model.

Version	The difference to the initial/preceding model (paths that been rejected)
i+1	P_KS_, P_SR,_ P_RK_, P_RA_, P_AS_
i+2	P_KA_, P_CK_
i+3	P_KC_, P_AC_, P_RA_
i+4	P_CA_, P_RC_

Source: Own study

In the next step, the path relationships between individual variables from the initial model plus economic efficiency were added to the model (adding them at the beginning was not possible due to the excess of parameters for the initial model in relation to the information contained in the correlation matrix). The aim of this was to answer the research question regarding the relationship between individual activities in the process of developing dynamic capabilities and the economic effectiveness of the organization. In the case of the variable that is economic efficiency, a one-way relationship was assumed, which means the influence of individual variables describing dynamic capabilities on efficiency.

**Fig 5:**
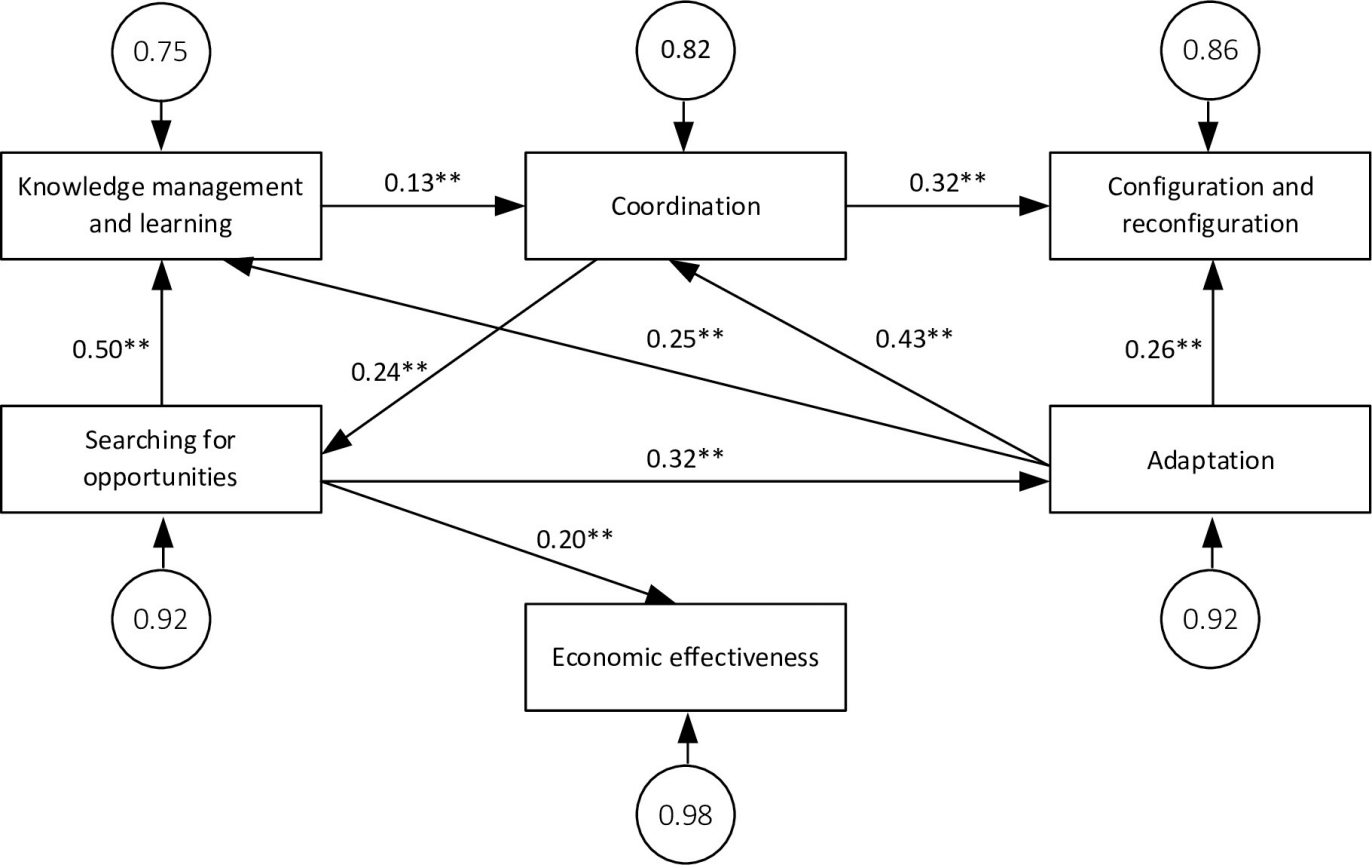
Results of SEM analysis. Source: Own study.

In this way the model shown in Fig 5 was obtained, for which the p-value was 0.45; SRMR was 0.027; with GFI NFI, CFI, and IFI being respectively: 0.995, 0.990, 0.998, and 0.998.

The results of the SEM analysis, presented in Fig 5, suggest the existence of positive links between the components of the process of developing dynamic capabilities. What is more, it can be observed that through mutual interactions and apparent feedback, it is possible to correct the actions at different stages in the process of developing dynamic capabilities.

In order to investigate the relationship between the overall level of dynamic capability development and economic effectiveness, a regression analysis was also performed for these variables (Table 5).

**Table 5:** Regression of the dependent variable–the economic effectiveness of the organization in relation to dynamic capabilities.

	R^2 = .04661		
	β	t-statistic	p
DC (HOI)	0.216	4.78856	0.000002

Source: Own study

For the performed regression analysis, the impact coefficient (beta) was 0.216 at a t statistic level of 4.78, and the p-value was below 0.001.

## Research discussion

Our research also suggests that activities carried out in the area of searching for opportunities have a positive impact on the level of economic effectiveness achieved by an organization, although the identified weak links between activities in the area of searching for opportunities and economic effectiveness (β = 0.199; p <0.00), suggest that searching for opportunities may be a necessary but not sufficient condition for such effectiveness. Moreover, on the basis of the research results one can formulate an observation about the moderating character of dynamic capabilities from the perspective of achieving economic effectiveness.

The research suggests the possibility of outlining a peculiar ‘path of developing dynamic capabilities’, whose beginning is marked by searching for opportunities activities, having a strong impact on knowledge management and learning (β = 0.50; p <0.00) and average adaptation (β = 0.32; p <0.00), as well as having a weak impact on the level of economic effectiveness achieved by organizations (β = 0.20; p <0.00), which indicates that managers should focus on improving activities in this area. Although the chronology of activities resulting from the model may seem contradictory to the logic of efficient operation [66] (research suggests that activities related to searching for opportunities precede activities related to acquiring knowledge), it is possible to point to premises justifying the observed order. The application of a different way of proceeding, in which the acquisition of knowledge preceded searching for opportunities, would force a necessity to acquire redundant information. In such a case the organization would have to bear the costs of acquiring and gathering redundant information that they would not be able to use, which from the business point of view would be difficult to justify.

The results of the study suggest that the implementation of projects within the framework of searching for opportunities allows activities related to the adaptation of the organization to be shaped to the changes in the environment. Although the strength of the impact is average, the rationality for these types of activities can be seen–respondents taking actions aimed at adapting to changes in the environment are guided by the previously identified opportunities, which indicates the importance of both the systemic approach to improving the organization [95] and analytical activities in the decision-making processes [121].

The adaptation to dynamic capability affects knowledge management and learning (β = 0.24; p <0.00) and, on average, coordination (β = 0.43; p <0.00) as well as configuration and reconfiguration (β = 0.26; p <0.00). While the link between adaptation and coordination as well as configuration and reconfiguration seems obvious–adaptation to changes in the environment allows the conditions of the organization to be shaped, and thus enables the management of dynamic capabilities [33], in the case of linking adaptation to knowledge management and learning, an impact in the opposite direction should be expected. The literature suggests that it is the appropriate knowledge resources to adapt to changes in the environment [42, 122]. In attempting to interpret the results obtained, it should be assumed that, similarly to the relationship between searching for opportunities and knowledge management and learning, the initial implementation of activities in the area of adaptation protects the organization from any appearance of the phenomenon of redundant information and skills. At the same time it should be noted that although no direct impact of knowledge management and learning on adaptation has been identified, it can be pointed out that there is an indirect impact, which is realized in a feedback loop through coordination and searching for opportunity activities. This state of affairs means it is worth addressing the issue of the experience curve [123]. For example, activities aimed at changing the business model or managing the organization’s borders force companies to take up activities in the area of acquiring new knowledge/learning.

The implementation of activities in knowledge management and learning processes affects coordination (β = 0.13; p <0.01), although the influence is small. The limited importance of knowledge and skills as a starting point for acquiring and securing resources and skills is puzzling in the light of the discussion in the literature on the subject, and undoubtedly it would be worthwhile to address these links more broadly in subsequent research on dynamic capabilities building processes.

The results of the research suggest that coordination has a weak impact on searching for opportunities (β = 0.24; p <0.00) and an average impact on configuration and reconfiguration (β = 0.32; p <0.00). The integration of activities in the organization, carried out in the area of coordination, ensures a faster flow of information and links between units in the organization dealing with the analysis of phenomena in the environment, thus allowing for the optimal use of emerging opportunities. At the same time, the average impact of coordination on configuration and reconfiguration indicates the importance of activities related to the acquisition and disposal of resources and skills to ensure appropriate conditions for the functioning and development of the organization.

In the light of the conducted research, it is puzzling that configuration and reconfiguration has no impact on other components in the process of developing dynamic capabilities. It should be expected that providing appropriate organizational solutions would enable better adaptions to changes in the environment, which was not confirmed in the research proceedings.

### Theoretical and managerial implications

This study contributes to theory and practice in several important ways. First, it extends knowledge in the area of dynamic capability development. The study presents the proposition of a proprietary model for the process of developing dynamic capabilities influencing the economic effectiveness of an enterprise, which includes 27 components covering five activities: searching for opportunities, knowledge management and learning, coordination, configuration and reconfiguration, and organizational adaptation. Although the issue of adaptation to environmental changes is at the heart of dynamic capabilities (Teece et al., 1997), the discussion on the essence of dynamic capabilities and their participation in strategic adaptation capabilities is still open (Suddaby, Coraiola, Harvey and Foster, 2020). In this context, this article provides new information on the configuration of the dynamic capability deployment process and sheds light on the activities that underlie dynamic capability formation.

Another contribution made by the study relates to the identification of links between activities in the model for developing dynamic capabilities. In previous studies, when describing the relationships between actions in models for developing dynamic capabilities, an a priori assumption was made about the linearity of actions, while the research we carried out suggests the existence of multilateral relationships between components in the process of developing dynamic capabilities (Fig 5).

The results of this article suggest that managers striving to develop dynamic capabilities should be aware of the importance of actions in the area of seeking for opportunities, which constitute the beginning of the "path of developing dynamic capabilities". Dynamic opportunity-seeking searches for opportunity capabilities include the following: (a) analysing trends and phenomena in the environment, aimed at creating new customer needs and anticipating the actions of competitors; (b) creating new ideas to mobilize participants and leading to changes in their attitudes; (c) identifying the changes needed as a result of phenomena occurring in the environment and the organization’s potential, affecting activities in the area of knowledge and learning management and in the area of adaptation, as well as the effectiveness of the organization. The results of the study suggest, therefore, the need for managers to clearly define mechanisms in the area of seeking for opportunities, allowing dynamic capabilities to be developed. It should be noted that our research suggests the critical importance of opportunity-seeking in developing dynamic capabilities, while previous research has ignored the impact of activities in this area. On the other hand, our research revealed a limited impact of activities in the area of configuration and reconfiguration on the remaining components of the process of developing dynamic capabilities, which were considered key in previous studies.

The study’s findings also shed light on the ways that enable managers to develop dynamic capabilities and the pattern of actions in developing dynamic capabilities. The links established by us, indicating the lack of linearity between actions in the process of developing dynamic capabilities, can be treated in terms of practical guidelines. Thus, the abstract concept of dynamic capabilities from the perspective of managers, thanks to the possibility of operationalization, acquires the value of applicability. The research does not provide conclusive evidence for linking dynamic capabilities with organizational effectiveness, suggesting that such dependencies may appear in the area of opportunity-seeking, although the identified links are not strong and require deepening of research.

### Conclusions and further research directions

The study deepens the knowledge about dynamic capabilities and the processes of their formation, and indicates the indirect impact of dynamic capabilities on the economic effectiveness of an organization. Using empirical research and structural equation modelling, this paper provides evidence of the links between individual components of dynamic capabilities and suggests the impact dynamic capabilities has, through activities searching for opportunities, on economic effectiveness.

However, it can be concluded from the conducted research that dynamic capabilities act as moderators on achieving effectiveness, rather than determining effectiveness per se. This state of affairs is consistent with other research results indicating the indirect impact of dynamic capabilities on corporate performance [53, 104, 117]. The direction of research on the interaction of dynamic capabilities with business results therefore requires further scientific exploration.

This proposal is an extension of the Zollo and Winter model [11] suggesting that dynamic capabilities include processes related to integration, reconfiguration, acquisition and the release of resources; and the Ambrosini and Bowman model [16] suggesting that dynamic capabilities include processes related to reconfiguration, use, learning and creative integration. However, unlike earlier studies, we suggest that there are multifaceted feedbacks between the components of the dynamic capabilities development process, and provide evidence for the importance of opportunity-seeking activities that can be considered critical to the dynamic capabilities process.

Research on the process of developing dynamic capabilities draws attention to further possibilities for scientific exploration. Although in the research procedure we adopted a five-stage model of developing dynamic capabilities, it is worth paying attention to the existence of various categories of processes affecting the dynamic capabilities of an organization (including closed and open processes). Thus, secondly, an interesting direction for research is the perspective of developing dynamic capabilities, taking into account the context of various groups of stakeholders influencing the dynamic capabilities of an organization. Thirdly, an interesting direction for research seems to be the importance of organizational knowledge and the experience curve in developing dynamic capabilities.
